# Novel Biologic Therapies for Thymic Epithelial Tumors

**DOI:** 10.3389/fonc.2014.00103

**Published:** 2014-05-07

**Authors:** Yuanbin Chen, Helen Gharwan, Anish Thomas

**Affiliations:** ^1^Thoracic and Gastrointestinal Oncology Branch, National Cancer Institute, National Institutes of Health, Bethesda, MD, USA

**Keywords:** thymic epithelial tumors, thymoma, thymic carcinoma, cixutumumab, sunitinib, belinostat

Thymic epithelial tumors (TETs) are comprised of a spectrum of histologically distinct tumors that also exhibit differences at the molecular level (1). Surgery is the mainstay of treatment but locally advanced and metastatic TETs can be inoperable and are associated with worse survival (2). Although multi-agent chemotherapy is associated with objective response rates (ORR) of 50–90% in the front-line setting [e.g., cisplatin, doxorubicin, and cyclophosphamide (CAP) (3), doxorubicin, cisplatin, vincristine, and cyclophosphamide (ADOC) (4)], no standard systemic treatments exist for relapsed or refractory TETs. Several biological agents have been evaluated in TETs in small phase II trials as illustrated in Table 1.

**Table 1:** **Published biological therapies in TETs**.

Trial	Agent	Target	*N*	ORR (%) (CR + PR)	TTP (months)	PFS (months)	Survival (months)
Palmieri et al. (5)	Octreotide/lanreotide ± prednisone	Somatostatin receptor	16	6 (37)	14	NR	15
Thymoma			10	4 (40)	NR	NR	NR
Thymic carcinoma			3	1 (33)	NR	NR	NR
Loehrer et al. (6)	Octreotide ± prednisone	Somatostatin receptor	38	12 (32)	NR	NR	NR
Thymoma			32	12 (38)	8.8	NR	Not reached
Thymic carcinoma			6	0	4.5	NR	23.4
Giaccone et al. (7)	Belinostat	HDAC	40	2 (5)	NR	NR	NR
Thymoma			24	2 (8)	11.4	NR	Not reached
Thymic carcinoma			16	0	2.7	NR	12.4
Thomas et al. (8)	PAC–belinostat	HDAC	13	7 (54)	NR	NR	NR
Thymoma			7	5 (71)	NR	NR	NR
Thymic carcinoma			6	2 (33)	NR	NR	NR
Rajan et al. (9)	Cixutumumab	IGF-1R	49	5 (10)	NR	NR	NR
Thymoma			37	5 (14)	9.9	NR	27.5
Thymic carcinoma			12	0	1.7	NR	8.4
Kurup et al. (10)	Gefitinib	EGFR	26	1 (4)	4	NR	NR
Thymoma			19	NR	NR	NR	NR
Thymic carcinoma			7	NR	NR	NR	NR
Bedano et al. (11)	Erlotinib + bevacizumab	EGFR	18	0	NR	NR	NR
Thymoma		VEGF	11	0	NR	NR	Not reached
Thymic carcinoma			7	0	NR	NR	Not reached
Thomas et al. (12)	Sunitinib	VEGFR	35	4 (11)	NR	NR	NR
Thymoma		PDGFR	16	1 (6)	NR	5.5	NR
Thymic carcinoma			19	3 (16)	NR	6.2	NR
Salter et al. (13)	Imatinib	KIT	11	0	NR	NR	NR
Thymoma		PDGFR	0				
Thymic carcinoma			11	0	NR	NR	NR
Giaccone et al. (14)	Imatinib	KIT	7	0	2	NR	4
Thymoma		PDGFR	2	0	8.5	NR	Not reached
Thymic carcinoma			5	0	1	NR	2
Palmieri et al. (15)	Imatinib	KIT	15	0	NR	3	Not reached
Thymoma		PDGFR	12	0	NR	NR	NR
Thymic carcinoma			3	0	NR	NR	NR
Wakelee et al. (16)	Saracatinib	SRC	21	0	NR	NR	NR
Thymoma			14	NR	NR	3.4	Not reached
Thymic carcinoma			7	NR	NR	1.4	Not reached

*NR, not reported*.

## Somatostatin Analogs

Somatostatin receptors are expressed in TETs and can be detected by octreotide scan (17). Palmieri et al. first showed efficacy of octreotide/lanreotide with or without prednisone in TETs (5). In another larger phase II trial, 38 patients with octreotide scan-positive TETs were treated with octreotide for 2 months. Responding patients continued to receive octreotide alone whereas patients with stable disease received additional prednisone for a maximum of 10 additional months. Two complete (5.3%) and 10 partial responses (25%) were observed in patients with thymoma, but no response was seen in thymic carcinoma (6).

## Histone Deacetylase Inhibitors

Histone deacetylases (HDACs) regulate gene expression through chromosome remodeling. Belinostat is a HDAC inhibitor that has been evaluated in a phase II trial in patients with advanced TETs after failure of platinum-containing chemotherapy (7). Among 25 patients with thymoma, and 16 with thymic carcinoma, two patients with thymoma achieved partial responses. No responses were seen among patients with thymic carcinoma. Median time to progression in patients with thymoma and thymic carcinoma was 11.4 and 2.7 months, respectively. Median survival was not reached in patients with thymoma and it was 12.4 months in patients with thymic carcinoma. Belinostat has also been evaluated with CAP in the front-line setting in a phase I/II trial. The overall response rate was 71% in thymoma and 33% in thymic carcinoma (8).

## Insulin-Like Growth Factor Receptor Inhibitors

Thymic epithelial tumors express insulin-like growth factor-1 receptor (IGF-1R), particularly recurrent or advanced tumors and those with aggressive histological subtypes (18). Cixutumumab, a fully human IgG1 monoclonal antibody that binds to IGF-1R with high affinity and induces internalization and degradation of the receptor, has been evaluated in a phase II trial of 37 patients with thymoma and 12 patients with thymic carcinoma, who had progressive disease after prior platinum-containing chemotherapy (9). Patients received cixutumumab at a dose of 20 mg/kg intravenously every 3 weeks until disease progression or development of intolerable toxicities. With a median follow up of 24 months, 5 of 37 thymoma patients achieved a partial response (ORR 14%). The median time to progression was 9.9 months and median survival was 27.5 months. In contrast, no responses were seen in patients with thymic carcinoma and the median time to progression and overall survival were 1.7 and 8.4 months, respectively. A significant increase in IFNγ-expressing CD4+ T cells and reduction in circulating endothelial progenitor cells (CEPs) were observed with treatment among responders. The potential predictive value of these biomarkers is under further investigation.

## Multikinase Inhibitors

Multiple case reports have described responses to the multikinase inhibitors, sorafenib (19) and sunitinib (20) in patients with previously treated thymic carcinoma. To confirm the activity of sunitinib in previously treated TETs, 22 patients with thymoma and 16 with thymic carcinoma with progressive disease following at least one platinum-based chemotherapy regimen were enrolled in a phase II study. Sunitinib was administered orally at a dose of 50 mg once daily in 6-week cycles (4 weeks on 2 weeks off). In 19 evaluable patients with thymic carcinoma and 16 evaluable patients with thymoma, the ORR was 16 and 6%, respectively, and PFS was 6.2 and 5.5 months, respectively. Adverse events included cytopenia, fatigue, mucositis, hypertension, and reversible decline in left ventricular ejection. Additionally, 10 (53%) patients with thymic carcinoma had tumor shrinkage between 10 and 29% (12).

## Epidermal Growth Factor Receptor, KIT and Src Inhibitors

Despites preclinical data demonstrating overexpression of epidermal growth factor receptor (EGFR) (21) and KIT (22) and Src Kinase (23) in TETs, very low objective responses were observed in phase II studies evaluating these agents. A study using EGFR inhibitor, Gefitinib, yields only one response in 26 patients (10) while another study using Erlotinib plus Bevacizumab showed no response (11). Three trials evaluating KIT inhibitor Imatinib in TETs (13–15) showed zero objective response. It is notable that although most tumors on these trials overexpressed KIT, no sensitizing mutation was found. Src inhibitor Saracatinib also failed to yield any objective response in a TET trial (16). For more details on these studies, see Table 1.

## Ongoing Trials of Targeted Therapies in TETs

Milciclib, an inhibitor of cyclin-dependent kinase 2/cyclin A complex and tropomyosin receptor kinase A (TrkA) is being evaluated in two phase II studies in patients with recurrent B3 thymoma and thymic carcinoma (NCT01011439 and NCT 01301391).

A phase II study is evaluating the role of the mammalian target of rapamycin (mTOR) inhibitor, everolimus in patients with TETs previously treated with chemotherapy. (NCT02049047).

## Conclusion

In conclusion, although the presence of targetable mutations detected by current techniques of molecular profiling is low, whole genome and exome sequencing has the potential to uncover novel biological targets. Although many trials of targeted agents have yielded disappointing results, the unprecedented activity of sunitinib in thymic carcinoma and the disease stabilizing effect of cixutumumab in thymoma provides an impetus to continue to explore the role of novel biological agents in TETs.

## Conflict of Interest Statement

The authors declare that the research was conducted in the absence of any commercial or financial relationships that could be construed as a potential conflict of interest.
